# Exploring Intraindividual Profiles for Home Buildings Based on Architectural Compositional Elements and Psychological Health Factors: A Transdisciplinary Approach

**DOI:** 10.3390/ijerph18168308

**Published:** 2021-08-05

**Authors:** Raquel Lara-Moreno, Ester Lara, Débora Godoy-Izquierdo

**Affiliations:** 1 Departamento de Psicología Social, Facultad de Psicología, Campus Universitario de Cartuja, Universidad de Granada, 18071 Granada, Spain; 2 Grupo de Investigación Psicología de la Salud y Medicina Conductual (CTS-267), Centro de Investigación Mente, Cerebro y Comportamiento, Facultad de Psicología, Campus Universitario de Cartuja, Universidad de Granada, 18071 Granada, Spain; 3 Escuela Técnica Superior de Arquitectura, Campo del Príncipe, Universidad de Granada, 18071 Granada, Spain; esterlara.arquitectura@gmail.com; 4 Departamento de Personalidad, Evaluación y Tratamiento Psicológico, Facultad de Psicología, Campus Universitario de Cartuja, Universidad de Granada, 18071 Granada, Spain

**Keywords:** psycho-architectural profiles, compositional elements, regulatory parameters, transactional perspective, salutogenesis, cluster analysis

## Abstract

Based on the transactional and salutogenic perspectives, we explored individual profiles that integrate psychosocial factors and compositional elements of the built home environment. Adults with different socio-demographic characteristics completed several self-report measures on psychological factors (personality traits, self-efficacy, mental health, and happiness) and architectural elements constituting the ideal home environment. Adopting an individual-centered perspective, three distinct intra-individual psycho-architectural (person-environment) profiles were found with different compositional preferences and psychosocial characteristics in terms of functioning, health, and well-being: endopathic (characterized by higher levels of psychosocial resources and well-being indicating a highly adapted and successful profile, and architectural preferences corresponding to their identities and experiences—expression through spaces), assimilative (characterized by average levels in all regulatory parameters indicating moderately adaptive individuals, and architectural preferences of spaces created in interactive processes—introjection of spaces), and additive individuals (characterized by a comparatively dysfunctional, poorer psychosocial profile, and architectural preferences in line with provoking a restorative effect—change with spaces). An awareness of the psychosocial features of the users for whom the homes are built can help in designing spaces to inhabit that are adapted to them for an enhancement of their overall well-being. Therefore, a better understanding of the interconnections between psychology and architecture will help in designing healthy spaces.

## 1. Introduction

Recently, greater importance has been given to investing in the health of the population through the design of supportive environments that can enhance health and well-being at all ages [1]. Thus, there is increasing interest in the influence of place on individuals’ health [2] and personal and social lives [3]. Place—as including physical-social, aesthetic-functional, and subjective-experiential-relational dimensions—is an experienced and socially constructed concept [4]. Psychology can make a valuable contribution by establishing how the mental states and behavior of individuals interact with the environment and by providing insights into the impact of urban, building, and residential systems on psychosocial functioning and mental health [5,6].

Awareness of how the built environment has an impact on well-being and health is a key factor when designing healthy places [7,8,9,10]. New transdisciplinary proposals have emerged that consider the potential for innovative architectural solutions for creating healthier built environments, such as the transactional perspective [11], which establishes the concept of person-environment, a global unit in which the individual and environmental variables are considered inseparable; the salutogenic design [12,13], according to which constructed buildings should be places that contribute towards improving health and a sense of well-being; and environmental psychology [14], providing an explanation of how the socio-physical environment can influence people’s functioning and well-being. Indeed, some experts state that human behavior is shaped by the environment [15,16,17]. Thus, compositional variables (architectural factors) and regulatory parameters (psychosocial factors) are interdependent and form a dynamic system that can be shaped by the individual.

There is a growing body of research on the link between architectural elements in work, educational, recreational, or health-care buildings and outdoor spaces/urban environments and the users’ psychological processes, functioning, and health (e.g., [2,18]), and on the relationship between places and recovery in individuals with mental health issues (e.g., [3,4,19,20,21]). Nonetheless, the link between architectural elements of residential or home buildings and the well-being of the general population has been largely unexplored (e.g., [22]). It has also been emphasized the need to distinguish between the influence of contextual (environmental level) factors and psychological (individual level) factors and their interactions, as well as to increase the quality of the research regarding these issues by incorporating interdisciplinary approaches [2]. Inspired by the transactional and salutogenic approaches, this study focuses on the relationships between individuals’ psychological factors and the features of the built environment as a home or intimate, indoor spaces from a cross-disciplinary perspective.

### 1.1. The Salutogenic or Psychosocially Supportive Design

Antonovsky’s salutogenic theory [23] was adapted by architect A. Dilani to promote mental health through the design of the physical environment (psychosocially supportive design). The main function of this type of design is to promote psychological processes that could bring about positive mental states, as well as eliminate or reduce emotional distress. Design from a salutogenic perspective not only considers the causes of stress but introduces wellness factors that strengthen health processes [24]. In identifying these factors that promote health, it is necessary to consider an individual’s personal and social resources, health, and sense of coherence, i.e., a global orientation that expresses a pervasive, enduring though dynamic feeling of confidence in personal controllability of the demands of the internal or external context. This sense of control is determined, in turn, by comprehensibility (i.e., the ability to understand what is happening in the environment and the course of events), meaningfulness (i.e., motivation to achieve goals in an integrated and meaningful life plan), and manageability (i.e., the ability to acquire and execute the resources required to manage the reality) [23].

Applied to architecture, the idea of Einfünhlung (endopathy) was derived from the factor of comprehensibility. Endopathy encompasses an aesthetic that turns the object into a symbol by projecting a feeling onto such an object [25,26], so that the use made of a constructed space allows the users to orient, identify, and recognize themselves within it [27,28,29,30]. In addition, the feeling also emanates from the object itself, which provokes positive or negative affective or empathic responses [31], giving rise to a certain level of mental well-being [32,33,34]. These affective reactions are due to the establishment of aesthetic rules based primarily on shapes (e.g., horizontal line: rationality; straight line: strength; curved line: flexibility; square: certainty, integrity; circle: balance, control) [30,35,36,37]. Based on these premises, it is concluded that aesthetics are not only visual but represent an intimate relationship between the mind and the built environment. This has provoked a radical change in architecture, giving it a new focus and allowing it to be understood as a dynamic relationship between the individual and the space in which place, rhythm, and time are found [38,39], so that the user is able to understand the built environment with which he or she interacts, promoting better mental health [32,40,41,42].

The meaningfulness factor gave rise to the concept of existential and *ideal* spaces, understood as living entities whose provoked emotions emanate from the movement of the user through the space. A dematerialization of the volumes is produced, which Wright ([43], pp. 141–142) calls the “destruction of the box”, whilst time acquires greater importance [44], and new materials appear (e.g., glass walls, metal columns), highlighting the journey as fundamental for understanding the building and how the habited spaces flow [45] (e.g., Le Corbusier, Frank Lloyd Wright, the Bauhaus, and the International Style) [46]. Space ceases to be fixed and immobile and acquires a relative value that depends on the user’s experience, which is contained within the space. The ideal space arises from the learnings, schemes, goals, and aspirations that people create for themselves during their interaction with the environment [27,43,46], giving rise to a process of assimilation (i.e., people accommodate to the existing space and try to integrate it into their mental schemes) or addition (i.e., people seek to find their own mental schemes in the architecture and create spaces that correspond to those experiences) [47]. This drive to create spaces that fit with the individuals’ existential experiences is satisfied through the manipulation of physical aspects, such as shape, gravity, and proportion-scale (horizontality, verticality) [48]. Thus, the human interior space, which is intangible and cannot be represented beyond one’s own imaginary, becomes the protagonist of architecture, since it translates those individual experiences into a three dimensional and geometric response ([49], pp. 13–15). In short, architecture is about creating spaces (meaningfulness) that evoke a feeling of functionality that is appropriate for the person (comprehensibility).

Following this premise, Kahn [50] emphasized the primary importance of *what* one seeks to create (immeasurable), as well as the idea that design is more concerned with *how* it is done (measurable), given the abstract nature of the former. He establishes that the how always responds to a what (will to be) that can only be achieved with an approach to feeling and a distance from thinking, but which in harmony give rise to the form (what) (e.g., “house”), which varies in terms of the interpretation of the design (how) (e.g., a house), giving the space its individuality and identity based on the experiences of the individuals within it. Those feelings and thoughts are of immeasurable value and are part of the mind; they provide the will to be and become measurable through a physical process [50]. Kahn’s proposals can be readily linked to the salutogenic theory developed by Dilani [24], in which the what corresponds to meaningfulness, namely, the aspirations that are sought to be achieved, that is, an ideal space of health and wellness, and the how corresponds to manageability, namely, the resources (compositional elements) that the architect has for creating such healthy spaces. Therefore, the manageability factor can be approached by considering the interactions between the multiple compositional elements and the various individual (regulatory) parameters. The study of the relationship between these variables as a whole allows for the creation of the ideal and healthy space.

These compositional elements have been profusely investigated (e.g., [24,32,33,34,41,42,51]), establishing the associations between them and the perceptions and experiences they evoke having a specific impact on mental health. According to Dietrich’s categorization of the compositional elements (i.e., shapes, lines, lighting, colors, materials, texture, mass, and space) [52], some research exists on simple shapes [53,54], complex shapes [35,36,55,56,57], organic shapes [40,58], color ranges [56,59,60,61,62], color saturation and intensity [63,64,65,66], light intensity [40,67], type of light [68,69,70,71,72], the openings [9,37,68,73,74,75,76,77], materials [61,78,79,80], order and rhythm [81,82], and mass [83].

### 1.2. Aims and Hypotheses

Based on these theoretical and applied concepts, this study aims to identify architectural configurations for home spaces that are differentially associated with psychosocial functioning. This will allow for identifying the patterns of relationships between these factors, which can then be used to create healthy domestic places where the user is included as a key element in the process. To this end, we aimed to identify compositional variables that are relevant to creating an ideal [24,50] domestic space, as well as to establish how these building-based elements interact with psychosocial variables regarding personality, self-confidence, mental health, and subjective well-being (regulatory parameters), with the ultimate aim of creating psycho-architectural profiles that help the architect to generate projects tailored to the clients’ experiences and needs.

We expected to identify different combinations of compositional variables and regulatory parameters. In particular, we expected to find at least two differentiated profiles: at one extreme, adults with a profile of good mental health and well-being (adaptive personality, high self-efficacy, absence of anxiety and stress, high levels of mental well-being, and happiness), and at the other extreme, adults with a profile of poor mental health and well-being (dysfunctional personality, low levels of self-efficacy, high levels of anxiety and stress, poor mental health, and low levels of happiness). We also anticipated the possible emergence of a third profile, characterized by average individuals with intermediate levels of the regulatory parameters. We based our expectations on the abundant psychological literature focused on multidimensional psychosocial profiles and their relationship with healthy functioning (e.g., [84,85,86,87]). Moreover, each of these profiles would be defined by a different configuration of the compositional variables, leading to characterizations easily acknowledgeable in terms of architectural styles. To our knowledge, this is the first study to integrate architectural elements in the configuration of psychosocial profiles. Bearing this in mind, we expected that the ideal space—one that promotes the development and health of the user—will be configured through the compositional elements corresponding to a profile of good mental health and well-being.

## 2. Materials and Methods

### 2.1. Participants

A total of 231 females and males aged between 18 and 70 years (M = 33.13; SD = 12.78) with varied socio-demographic characteristics (Table 1) voluntarily participated in the study. The participants were recruited from the general population through several procedures (see below), forming a convenience sample of the entire national territory. Of all the individuals who accessed the online survey (N = 372), 37.9% did not meet the inclusion criteria, namely, being 18 years old or over, living in Spain, and completing the survey. As we expected that everybody can have an idea of his or her ideal home space, irrespective of gender, health status, socioeconomic status, relationship status, and so forth, and as we aimed to explore global profiles based on a range of psychosocial features to be representative of the diversity of individuals in the community, no strict exclusion criteria were adopted besides age and location.

The sample size was estimated prior to the study using the Clinical and Translational Science Institute (University of California, San Francisco) online calculator for correlational research [88] in 194 participants for *alpha* = 0.05, *beta* = 0.02, and *r*s for several associations among the study variables previously reported (estimated average *r* = 0.20) (e.g., [89,90,91]). We decided to recruit as many individuals as possible above this number.

### 2.2. Measures

The online survey included the following measures:

Big Five Inventory (BFI-10) [92], short version [93]. This inventory evaluates personality traits across five dimensions: extraversion (outgoing/energetic vs. solitary/reserved), neuroticism (sensitive/nervous vs. resilient/confident), openness to experience (inventive/curious vs. consistent/cautious), agreeableness (friendly/compassionate vs. challenging/callous), and conscientiousness (efficient/organized vs. extravagant/careless). Participants respond on a Likert-type scale (1 = Totally agree, 5 = Totally disagree) to 10 statements, two for each dimension. Five partial scores are obtained by addition. The BFI-10 has psychometric properties that are comparable to those of the original BFI [92]. This questionnaire has been used with the Spanish population in contexts related to health [94] and the environment [95]. Subscales Cronbach’s *alpha* ranged from 0.62 to 0.66 in the present study (note that each dimension is composed of two items).

General Self-Efficacy Scale (GSE) [96], Spanish version [97]. Based on Bandura’s [98] concept of self-efficacy, this scale measures perceptions of personal competence to effectively handle a wide variety of situations. The scale consists of 10 items with a Likert-type response format (1 = Disagree, 4 = Agree), and a total score was calculated by summing the scores obtained on each item. Its psychometric properties have been established in the Spanish population [97]. Cronbach’s *alpha* was 0.87 in the present study.

General Health Questionnaire-28 (GHQ-28), Spanish version [99]. This questionnaire has been widely used for mental health screening and is composed of 28 items that evaluate four areas of health and functioning (i.e., physical state, absence of anxiety, daily functioning, and absence of depression). The person responds by taking into account the previous four weeks (0 = No more than usual to 3 = Much more than usual). Four partial scores are obtained corresponding to each one of the subscales, along with a total sum score. Its psychometric properties have been established in the Spanish population [100]. Cronbach’s *alpha* was 0.90 in the present study.

Subjective Happiness Scale (SHS) [101], Spanish version [102]. It measures subjective well-being through four questions with a Likert-type response format (1 = Very unhappy, 7 = Very happy). This instrument provides an overall measure of subjective happiness which evaluates a molar category of well-being as a global psychological phenomenon, considering the definition of happiness from the respondent’s perspective. A global score was obtained. Its psychometric properties have been established in the Spanish population [102]. Cronbach’s *alpha* was 0.82 in the present study.

**Figure 1:**
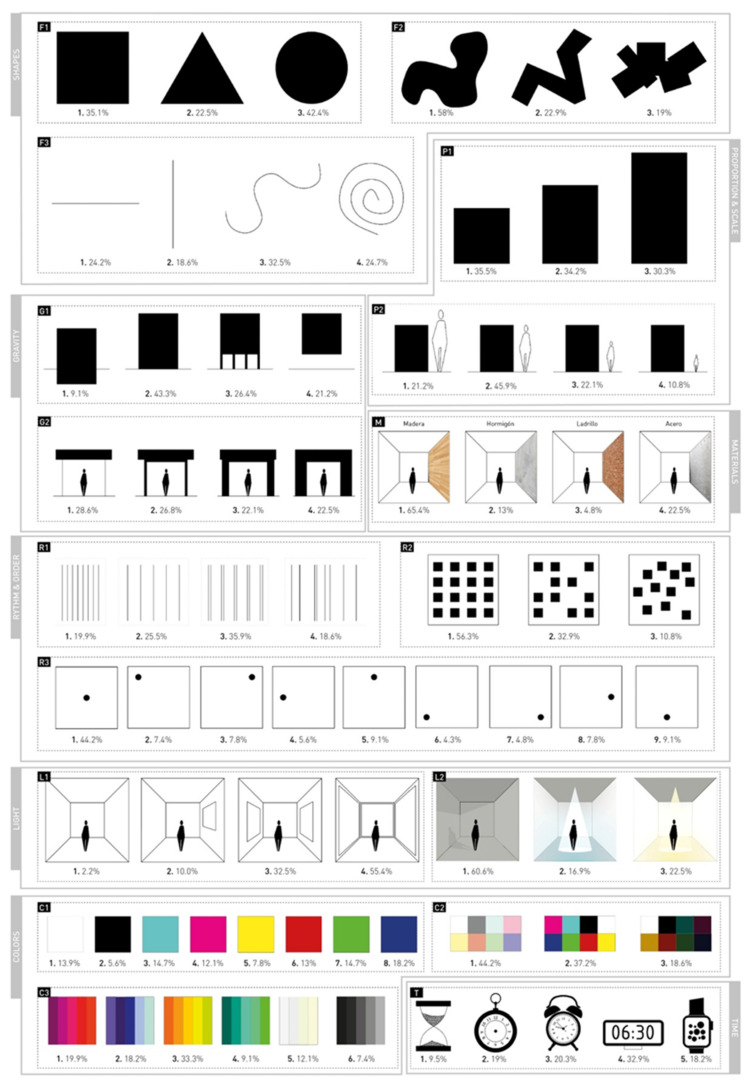
Frequencies and percentages of response on the CPIS.

Compositional Preferences for Ideal Spaces Scale (CPIS). This self-report was designed by the researchers specifically for the purposes of the present study. It is composed of a series of 17 groups of shapes and images that represent different concepts and compositional elements grouped into the following categories: (1) shapes, (2) proportion and scale, (3) gravity, (4) materials, (5) rhythm and order, (6) lighting, (7) color, and (8) time (as related to a historical period and its aesthetic style). The respondent indicates which figure in each category is preferred for an ideal domestic space in which to live and develop as a person. Each figure-variant is assigned a number which indicates the preferred compositional characteristic (see Table 2 and Figure 1). Due to the features of this survey-based tool, psychometric properties were not explored.

In addition to the above measures, participants answered questions regarding socio-demographic and personal data (age, sex-gender, relationship status, educational level, employment status, and level of monthly family income).

### 2.3. Procedure

The participants were recruited through the use of different social resources where the study was publicized (e.g., advertising in faculties and university schools, social networks and forums, mailing lists, and mobile communication apps) and word-of-mouth procedures, forming a convenience sample. All participants were provided with general information about the main objective of the study and were asked to participate voluntarily. Those who decided to participate received detailed information about the study and specific instructions on how to complete the questionnaires through the LimeSurvey^®^ platform and signed a consent form on the front page of the online survey. The measures were automatically counterbalanced to avoid order bias. The assessment could be completed by each participant in a single session, or the answers could be saved to be retrieved and continued at another time with a personal password.

### 2.4. Study Design and Statistical Analysis

This was a cross-sectional descriptive study. Preliminary analyses were conducted to detect and correct possible errors in the input of data, lost or absent data, extreme data or outliers, as well as to check parametric assumptions and make decisions about the statistical tests to be used. Due to the characteristics of the data, parametric tests were conducted for statistical analysis.

In addition to descriptive analyses (n and percentages for categorical variables, mean and standard deviation for continuous variables), correlation analyses were conducted using Pearson’s *r*-test and cluster analyses [103,104] were carried out using the hierarchical agglomerative method as an exploratory analysis, along with the non-hierarchical k-means algorithm as a definitive analysis. These analyses aimed to identify the psycho-architectural profiles maximizing intra-conglomerate homogeneity and between-conglomerate heterogeneity, complemented by a discriminant analysis. The optimal number of clusters was decided by means of the Pseudo F (PSF) criterion or variance ratio. In addition, Goodman and Kruskal’s *λ* values and the percentage of cases correctly classified were also considered. To establish possible differences between groups, a between-group ANOVA with post hoc *t*-Student pairwise comparisons (with correction for non-equal variance when necessary) were conducted. All scores on psychosocial variables were transformed into Z-scores [105].

The significance level for all analyses was set at *p* < 0.05. All of the analyses were conducted using the SPSS statistical package for Windows 23.0 (SPSS IBM Corp, Armonk, NY, USA).

## 3. Results

The descriptive results for the compositional variables are presented in Figure 1. The “ideal space”, i.e., the most chosen elements, was composed of the following: circular, curved-line, and organic geometric shapes; deconstructed or lightweight structures in any proportion, at human scale, and in contact with the ground; highly ordered structures of constant rhythm with duplicity and centered elements; very open spaces with daylight; wood as the predominant material; light colors of warm ranges; and modern elements.

Table 3 shows the descriptive results for the psychosocial variables. Participants obtained, on average, moderate-high scores for extraversion, openness, agreeableness, conscientiousness, general self-efficacy, mental health, and happiness, and moderate scores for neuroticism.

Pearson’s correlations (Table 3) indicated that the compositional variables associated with the regulatory parameters were complex geometric shapes (S2), rectangular proportions (P1), gravity-weight of the structure (G2), rhythm-position of elements in relation to each other (R2), rhythm-position of an object with respect to the space in which it is located (R3), daylight-openings (L1), and color range (C3). Almost all psychosocial variables were associated with a compositional variable. All significant correlations were in the expected positive or negative direction.

A k-means cluster analysis was conducted for fixed solutions between two to four clusters. In these analyses, the regulatory parameters considered were the five personality traits, general self-efficacy, overall mental health, and happiness. All of the compositional variables were included since, as architectural elements, these are all physically present and cannot be suppressed (e.g., any house must have a structure (gravity) and is built using certain materials, colors, shapes, distribution, proportion, and so forth). A three-cluster solution was chosen because this was the solution with the highest percentage of participants correctly clustered in each group and was supported by the PSF and *λ* values, which reached optimal values for the three-cluster solution. In addition, this solution has greater parsimony and replicability and can be more readily interpreted in a meaningful way. An initial ANOVA revealed significant differences between the clusters for all compositional variables, with the exception of pure (S1) and linear geometric shapes (S3), rectangular proportions (P1), materials (M), order of the successions of equal elements (R1), individual color (C1), and time (T) (*p* > 0.05). These variables were excluded for the final formation of the clusters.

**Figure 2:**
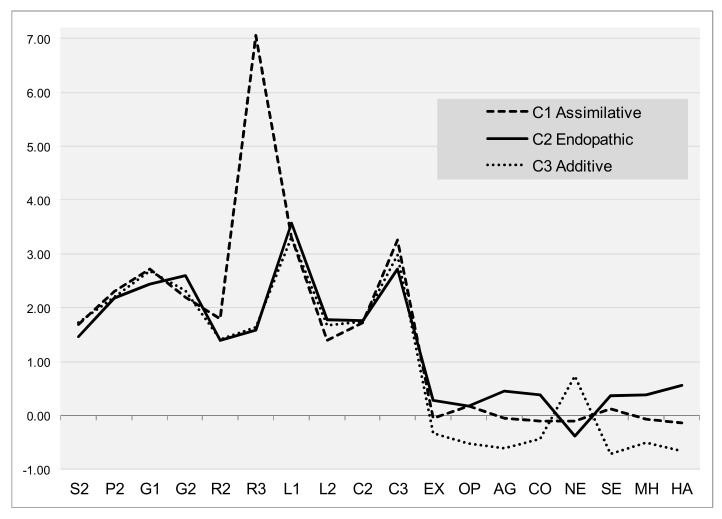
Graphical representation (means) of the psycho-architectonic profiles identified in the cluster analysis. S2 = Complex geometry; P2 = Proportion-human scale; G1 = Gravity-ground; G2 = Gravity-perceived weight; R2 = Rhythm-order; R3 = Rhythm-position; L1 = Lighting-openings; L2 = Lighting-daylight; C2 = Color-intensity and saturation; C3 = Color-range and temperature; EX = Extraversion; OP = Openness; AG = Agreeableness; CO = Conscientiousness; NE = Neuroticism; SE = General self-efficacy; MH = Global mental health; HA = Happiness.

Each configuration of the three definitive clusters identified was characterized by different psycho-architectural profiles (see Figure 2): Cluster 1, composed of 82 adults (35.5% of participants), was characterized by levels close to the average in all regulatory parameters (note that Z scores were used) and architectural variables highlighting the choice of angular, sharp spaces, moderately open spaces with natural lighting and warmer colors, lightweight structures on pilotis, and slightly ordered spaces with objects at the bottom of the space. This cluster was, therefore, referred to as the assimilative group. Cluster 2, composed of 90 adults (38.9%), was characterized by showing higher levels of extraversion, agreeableness, conscientiousness, openness, general self-efficacy, overall health, and happiness, along with low levels of neuroticism. With regard to architectural elements, the individuals of this cluster chose organic shapes, rigid structures on the ground, ordered spaces with objects at the top, very open spaces, and cold light with fewer warm colors. This cluster was, therefore, referred to as the endopathic group. Cluster 3, composed of 59 adults (25.5%), was characterized by high levels of neuroticism and low scores on extraversion, agreeableness, conscientiousness, openness, general self-efficacy, general health, and happiness. With regard to architectural variables, they chose a combination of elements of Clusters 1 and 2: they were similar to Cluster 1 in terms of shapes, gravity, openings, and color range; similar to Cluster 2, Cluster 3 also showed a preference for ordered spaces with objects at the top, primarily with non-natural, and cold light. Given that Cluster 3 differs primarily in terms of regulatory parameters, this group was referred to as the additive group. Discriminant analysis revealed a Wilks’ *λ* value of 0.532 (*Χ*^2^ = 138.626, *p* = 0.000) for the overall model, which indicates high discriminant power. Thus, 97.8% of the cases were correctly classified. Based on these profiles, we observed distinct architectural styles associated with different levels of adaptation, well-being, and health.

Further, the clusters were compared with respect to the set of psycho-architectural indicators using a one-factor ANOVA and pairwise comparisons. Significant differences were found between the clusters for all compositional variables except for complex geometric shapes (S2), perception of space-to-ground contact (G1) and weight (G2), and color range (C3), for which the differences were marginally significant. Non-significant differences were found for relative proportion to human scale (P2) and color intensity (C2) (Table 4 and Table 5). Bonferroni or Games-Howell pairwise comparisons, according to Levene’s *F*, indicated that there were significant differences (*p* < 0.05) between Cluster 1 and Cluster 2 in the regulatory parameters of agreeableness, conscientiousness, mental health, and happiness, and marginally significant differences for extraversion (*p* = 0.09). For the compositional variables, these clusters differed in terms of structure weight (G2), order of elements (R2), position of an object with respect to the space in which it is located (R3), daylight-openings (L1), artificial light (L2), and color range (C3) (*p* < 0.05). Additionally, significant differences were found between Cluster 1 and Cluster 3 in all the regulatory parameters (*p* < 0.05), except extraversion and consciousness (*p* > 0.05). However, only the differences in the compositional variables of order of elements (R2) and position of an object with respect to the space in which it is located (R3) were significant (*p* < 0.01). Finally, significant differences were found between Cluster 2 and Cluster 3 for all the regulatory parameters (*p* < 0.01), but none of the compositional variables.

## 4. Discussion

The present study had three objectives. First, we sought to identify the most preferred architectural configuration for the ideal domestic space. Second, we aimed to explore the associations between architectural components of such an environment and certain regulatory parameters in order to establish, as a third objective, different intra-individual configurations based on distinct psychosocial variables and architectural components, exploring the differences between the resulting psycho-architectural profiles.

The results suggest that the ideal domestic space is composed of a combination of circular (S1.3), organic (S2.1), and curved (S3.3) shapes, which is in line with findings previously reported [35,36,56,57], with less sharp objects being preferred over very angled objects. Shapes are, in turn, applied on a human scale (P2.2), developing as volumes that are in contact with the ground (G1.2), but deconstructed (G2.1). Although choosing a volume that is heavy, in contact with the ground and simultaneously light (deconstructed) might be seen as contradictory, this is not the case, since, as Arnheim [83] and Lipss [30] indicated, perception plays an important role in the understanding of the environment but, at a perceptual level, gravity is not attributed to weight. These choices appear to support the new ways of understanding architecture postulated by the International Style architects, in which they break with the old style and “destroy the box” and incorporate the concept of time into architecture, and with it the user and his or her motivations [43]. In addition, this deconstruction translates into greater openings and a consequent visual connection with nature and the entry of natural light, two elements that the salutogenic model highlights as indicators of well-being (e.g., [12,13,24,51,75,106]). Thus, the preferred lighting is daylight (L2.1) in abundance (L1.4), a finding supporting that obtained by Hesen and Lamberts [37] in their simulation study. This positive influence on health is also mentioned by other authors [32,40,74,75,76]. With regard to rhythms, the elements that constitute the ideal domestic space are ordered (R2.1) and centered (R3.1), as Makin et al. [81,82] stated when claiming that visual symmetry is closely linked to beauty and generates positive stimuli.

In terms of materials and colors, the ideal space is composed of warm materials of natural origin such as wood (M.1) and warm colors (C3.3), yet the choice of cold (C3.2) and desaturated (C2.1) colors is also notable, as seen in works such as those of Wright, Aalto, or the Eames. This duality is likely to be a consequence of the perception of wood as something associated with the home [50,61,79,80] along with the capacity of light and cold colors to induce calm and safety and simultaneously a sense of width [56,59,60,61,62].

In addition, we found a relationship between some of the compositional (physical) elements and the regulatory (psychological) variables. In particular, almost all the regulatory parameters correlated with one or more of the compositional elements. Specifically, the extraversion trait correlated negatively with non-pure geometric shapes (S2) and positively with daylight (L1); the openness trait correlated positively with rhythm-order and position (R2, R3); the consciousness trait correlated positively with rectangular proportion (P1) and gravity-weight (G2); the neuroticism trait correlated positively with non-pure geometric shapes (S2); and happiness correlated negatively with non-pure geometric shapes (S2) and color ranges (C3), and positively with daylight (L1). Other authors have found similar associations between these regulatory parameters and compositional elements (e.g., [24,32,33,34,41,42,51]). These results support the proposed importance of taking into account the relationship between regulatory parameters and compositional elements in order to plan spaces that promote well-being and health.

Bussery et al. [84] stated that one of the priorities in research related to subjective well-being is to better understand how various psychosocial configurations relate to different discriminant variables in order to inform the conditions necessary to improve wellness. Our results contribute to this objective, since we have identified three different intra-individual configurations each characterized by distinct architectural profiles that are uniquely linked to well-being and mental health indicators.

**Figure 3:**
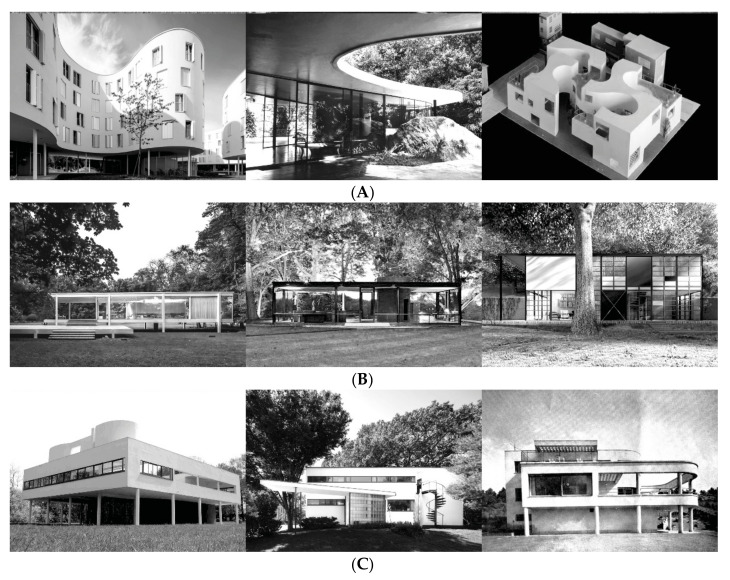
Panel (**A**): (From right to left) Apartments on Ave. Maréchal Fayolle, SANAA (2018); Das Canoas House, Oscar Niemeyer (1951); Okurayama Apartments, SANAA (2008). Panel (**B**): (From right to left) Farnsworth House, Ludwing Mies Van der Rohe (1946); The Glass House, Philip Johnson (1949); The Eames House, Charles and Ray Eames (1949). Panel (**C**): (From right to left) Villa Savoye, Le Corbusier and Pierre Jeanneret (1929); The Gropius House, Walter Gropius (1988); Villa Jaritz, József Fischer (1942).

One of the groups, which we have termed “endopathic”, is characterized by individuals who psychologically could be regarded as highly adapted and successful in comparison with the other subgroups, with greater levels of extraversion, conscientiousness, agreeableness, and openness, more self-efficacy, greater general health and happiness, and low neuroticism. This cluster represents an example of well adapted and happy people and is compatible with the profiles of successful people found at the psychosocial level in other classifications (e.g., [85,107]). With respect to the architectural components, these people prefer organic shapes (S2.1), cold lighting (L2.2) in abundance (L1.4), rigid volumes (G2.3) on the ground (G1.2), and ordered (R2.1) and dynamic spaces (R3.2) with a color range that has less warmth (C3.3). These choices appear to be more compatible with the architecture proposed by Sanaa or Niemeyer (Figure 3, Panel A). It is possible that the psychological traits of these individuals underlie their preferences for these spaces, which serve as a way to express or project their identity and experiences when creating or inhabiting the places, something that future research should explore more deeply.

A second group was composed of moderately adaptive and “assimilative” people. These individuals benefit from average mental health and their psychological resources work together to create a moderately desirable developmental process. At a psychological level, the profile of this group is compatible with those reported in previous studies (e.g., [85,107]). At the architectural level, there is a notable preference for slightly ordered (R2.2) and moderately open spaces (L1.3), day lighting (L2.1), sharp organic shapes (S2.2), and volumes that are weightless (G2.2) on pilotis (G1.3), with objects at the bottom of the space (R3.7), and warmer color ranges (C3.3). The architectural preferences of this profile correspond to the International Style [108], as represented by the work of Mies Van der Rohe, Philip Johnson, and Schinder (Figure 3, Panel B). These individuals introject the spaces, and, thus, their psychological states and behaviors are shaped by the places they inhabit.

A third group represents those people who are highly “dysfunctional” at a psychological level in comparison with the other subgroups. These individuals present high levels of neuroticism, less self-confidence, and low levels of adaptive personality traits, along with poor mental health and low levels of happiness. This cluster is compatible with the psychological profiles previously identified by Smith and Baltes [107] and Gerstorf et al. [85]. However, in spite of the psychological differences shown by this cluster, the preferred compositional elements were similar to those indicated by the assimilative group, except for the type of light, since this group prefers less natural light and cold artificial illumination (L2.2), in addition to showing a preference for very ordered spaces (R2.1), and objects positioned at the top of the space (R3.2). This architecture, also characterized by the International Style, is represented by architects such as Le Corbusier, Fisher, or Gropius (Figure 3, Panel C). Fortunately, this group had the least number of participants. Perhaps these people prefer the environments chosen by psychologically more functional people in order to achieve better levels of well-being. In other words, places that they create and inhabit are neither an indication of how they are, feel, and behave (as in the case of the endopathic cluster), or a consequence of interacting with the spaces (as in the case of assimilative individuals), but are instead an expression of what they are looking for with such spaces, that is, such people have an “additive” profile. The differences found with respect to the previous cluster may reveal, however, their true dysfunctional psychological profile. These explanatory hypotheses are only speculative and need to be confirmed by further research.

The clusters differed significantly in terms of psychosocial configurations. In terms of architectural elements, whereas the endopathic and assimilative profiles differed markedly from each other, the additive subgroup was very similar to both of these aforementioned profiles. Consequently, the regulatory parameters (but also some of the architectural components, particularly lighting and rhythm) seem to better discriminate the profiles found. These findings are in line with the results reported by other authors such as Hesen and Lamberts [37,68,72] in terms of lighting and Makin et al. [81,82] in terms of rhythm.

On the basis of the findings presented here, a series of practical applications can be derived that go beyond the conventional applications that focus mainly on clinical, recreational, work, and educational indoor environments. In particular, our findings could help to inform the design and creation of healthy residential environments, which include not only housing but can also be extrapolated to urban planning [109]. Thus, two main areas of application are proposed. For housing, this study suggests that the users (clients) can be included as the primary component of the design, providing the architect with the appropriate tools for creating a space that is adapted to them and their psychosocial needs—not only those of a physical-functional nature. For urban planning, conducting a study with a large population-based sample could help to create urban environments that have a positive impact on the mental health of their inhabitants. In addition, as multi-sensory environments are gaining popularity, our methodology and findings are useful also to assess the influences of multi-sensory spaces on psychological phenomena, and to use this information in turn to design indoor evidence-based spaces. Yet, multi-sensory rooms have usually been used as contextual spaces for rehabilitation (e.g., dementia, severe mental illness, developmental disabilities), there is a growing interest in exploring these spaces (e.g., artistically designed multisensory environments) in otherwise healthy individuals and their restorative influences on affective states [110,111]. As an innovative line of inquiry, we encourage to expand this research to architectural elements and building facets due to its potential clinical and non-clinical applications.

Despite the novelty and utility of our findings, this study presents some limitations that should be addressed in future research. First, the main limitation is the small sample size and its low representativeness in terms of sociodemographic variety, which limits the generalizability of the findings. Second, this is a self-report-based study, and although self-reports are considered appropriate for evaluating psychosocial variables, it would be desirable to confirm and complement the information collected with them by using other types of measures (e.g., medical reports), which are not subject to social desirability, memory, or response biases. The underlying anatomical substrates of mental processes [112,113] also allow for the use of other psychophysiological and neurophysiological measures. Moreover, there is a lack of standardized architectural-compositional measures, and more research is needed to validate the CPIS, created for this study, which is another reason to interpret the current findings with caution. Additionally, there is a need to continue investigating other aspects of the composition of spaces that could be of relevance (e.g., acoustics-noise, temperature-thermal comfort, natural green-blue elements, ventilation-air quality) [9]. Third, only some regulatory parameters have been included in our study, while many others have been ignored (e.g., quality of life, life satisfaction, and stress). Future research should include a wide range of cognitive, motivational, emotional, and behavioral factors. All of this could help the architect to design the spaces in a way that fits the client’s needs, desires, and expectations. Only by understanding how compositional variables interact with mental functioning to form psycho-architectural profiles (such as those revealed in this study), can places be designed that help people feel better and more complete in those spaces.

Fourth, we have not taken into account the possible impact of sociodemographic variables. Empirical evidence indicates that the influence of age, gender, socioeconomic level, or health status can be notable [114,115,116,117], and future analyses should aim to more rigorously control for their effects by including such variables as covariates in the analyses, or alternatively, explore the influence of these variables as moderators. Similarly, cultural influences [118,119,120] should be explored with research conducted in other countries and cultures and with diverse races/ethnicities. Finally, future research should aim to establish whether the intra-individual psycho-architectural profiles show relationships with external criteria variables that act as predictors, correlates, or consequences of such profiles, which would allow for the cross-validation of the uniqueness of the groups, since it is expected that different profiles will show unique relationships with outcome variables [107].

In spite of these limitations, our results are novel and interesting. These findings highlight the relevance of addressing regulatory parameters from the initial phases of the architectural project. Our findings also emphasize the importance of understanding the relationships between psychology and architecture, specifically in terms of architectural composition. By identifying the regulatory parameters that interact with compositional variables along with understanding their effects, we can increase scientific knowledge about the relationship between the fields of psychology and architecture. This is of considerable significance for informing the design and creation of built spaces and environments that are adapted to each individual. Moreover, it has been observed that the compositional elements of spaces—particularly rhythm, lighting, shapes, gravity, colors, and proportion—correlate with different psychological variables, which has allowed us to identify distinct psycho-architectural profiles. Thus, it will be necessary to use specific evaluation instruments that are sensitive to the experiences and needs of the target population in order to provide the architect with information that is accurate and useful. In this study we have used a measure that could represent a preliminary step forward in this regard.

## 5. Conclusions

To conclude, based on the transactional and salutogenic perspectives as theoretical frameworks and focusing on the ideal home as a built physical environment, we have found three distinct psycho-architectural profiles in the adult population, each of which reflect different configurations of wellness at emotional, functioning, and health levels. Our findings suggest that conducting a psychological analysis prior to the planning of an architectural project could be of considerable value for identifying the compositional variables associated with these regulatory parameters. By addressing all these factors, the users for whom the homes are built can inhabit spaces that are adapted to them, generating, as a consequence, an enhancement of their overall well-being and mental health. In turn, this would facilitate self-development, greater productivity, and other positive aspects from which they and the whole community can benefit. Finally, it is also worth mentioning the value that this architectural work could have for society in general, since factors such as mental health and subjective well-being can help to enhance life satisfaction, quality of life, and healthy life expectancy.

## Figures and Tables

**Table 1:** Sociodemographic data of the participants.

		N	%
Age	18–29 yr.	121	52.4
	30–49 yr.	73	31.6
	>50 yr.	37	16.0
Sex-gender	Female	154	66.7
	Male	77	33.3
Relationship status	Single	82	35.5
	Non-stable relationship (<1 year)	10	4.3
	Stable relationship (≥1 year)	134	58.0
	Separated-divorced-widow	5	2.2
Educational level	Primary	4	1.7
	Secondary	15	6.5
	Professional training	32	13.9
	University	180	77.9
Work status	Student	98	42.4
	Employed	110	47.6
	Unemployed	12	5.2
	Homemaker	6	2.6
	Retired	5	2.2
Family monthly income	<2000 €	104	45.0
	>2000 €	127	55.0
Nationality	Spanish	215	93.1
	Non-Spanish residing in Spain > 1 yr.	16	6.9
Location	East Andalucía	151	65.4
	Other	80	34.6

**Table 2:** Categories and subcategories of compositional elements in the CPIS.

Category	Subcategory	Figures and Assigned Values
Shapes (S)	S1. Simple geometry	Square (1) Triangle (2) Circle (3)
	S2. Complex geometry	Organic shapes (1) Sharp organic shapes (2) Complex shapes by addition (3)
	S3. Linear geometry	Horizontal line (1) Vertical line (2) Curved line (3) Spiral line (4)
Proportion and scale (P)	P1. Rectangular proportion	Square proportion (1) Golden proportion (2) Root proportion (3)
	P2. Proportion relative to human scale	Small scale (1) Human scale (2) Domestic scale (3) Large scale (4)
Gravity (G)	G1. Perception of gravity of the space with respect to the ground	Sunk in the ground (1) On the ground (2) On pilotis (3) Floating (4)
	G2. Perception of the structure weight	Deconstructed structure (1) Lightweight structure (2) Rigid structure (3) Mass structure (4)
Materials (M)		Wood (1) Concrete (2) Brick (3) Steel (4)
Rhythm and order (R)	R1. Succession of equal elements	Very frequent rhythm (1) Spaced rhythm (2) Constant rhythm with duplicity (3) No order (4)
	R2. Position of a set of elements in relation to each other	Ordered space (1) Slightly ordered space (2) Disordered space (3)
	R3 Global position of an object with respect to the whole space	Centered (1) Top left (2) Top right (3) Left centered (4) Top centered (5) Bottom left (6) Bottom right (7) Right centered (8) Down centered (9)
Lighting (L)	L1. Natural daylight/openings	Closed space (1) Slightly open space (2) Moderately open space (3) Very open space (4)
	L2. Artificial light/temperature of light	Daylight (1) Cold light (2) Warm light (3)
Color (C)	C1. Individual color	White (1) Black (2) Cyan (3) Magenta (4) Yellow (5) Red (6) Green (7) Indigo (8)
	C2. Color saturation/intensity	Bright color (1) Intense color (2) Unsaturated color (3)
	C3. Color range/temperature	Warm/red (1) Cold/blue (2) Warm/yellow (3) Cold/green (4) Neutral/white (5) Neutral/black (6)
Time (T)		Archaic (1) Ancient (2) Recent past (3) Modern (4) Contemporary (5)

**Table 3:** Descriptive findings for psychosocial variables and correlations with architectural elements.

Variable (Possible Range of Scores)	M	SD	S1	S2	S3	P1	G1	G2	R1	R2	R3	L1	C2	C3	T
Extraversion (2–10)	6.48	1.97		−0.17								0.14			
Neuroticism (2–10)	5.97	2.07		0.15							*−0.13*				
Openness (2–10)	7.81	1.78								0.15	0.13				
Agreeableness (2–10)	6.74	1.56				*0.11*	*−0.13*		*−0.12*						
Conscientiousness (2–10)	7.48	1.74				0.14		0.17		*−0.12*					
Self-efficacy (10–40)	28.77	5.04											*0.11*		
GHQ_Total score (0–84)	62.07	11.49													
Happiness (4–28)	20.97	4.53	0*.13*	−0.15	*0.12*				−0*.11*			0.28		−0.13	−0*.11*

S1 = Simple geometry; S2 = Complex geometry; S3 = Linear geometry; P1 = Rectangular proportion; G1 = Gravity-ground; G2 = Gravity-perceived weight; R1 = Rhythm-succession; R2 = Rhythm-order; R3 = Rhythm-position; L1 = Openings; C2 = Color-intensity and saturation; C3 = Color-range and temperature; T = Time. Regular font: *p* < 0.05, italic font: *p* < 0.10.

**Table 4:** Means (centroids), standard deviations, and between-group comparisons for psychosocial variables (Z scores) (N = 231).

Regulatory Parameters	Assimilative N = 82		Endopathic N = 90		Additive N = 59		*F*	*p*
	M	SD	M	SD	M	SD		
Extraversion	−0.056	1.001	0.272	0.965	−0.337	0.950	7.155	0.001 **
Neuroticism	−0.105	1.023	−0.385	0.846	0.733	0.784	28.437	0.000 **
Agreeableness	−0.050	1.006	0.448	0.740	−0.615	1.006	24.411	0.000 **
Conscientiousness	−0.108	1.005	0.382	0.915	−0.433	0.912	14.007	0.000 **
Openness	0.182	0.981	0.182	0.902	−0.530	0.996	12.210	0.000 **
General self-efficacy GHQ Total score Happiness	0.121 −0.063 −0.133	0.866 1.016 0.994	0.363 0.383 0.558	0.898 0.711 0.687	−0.722 −0.496 −0.667	0.962 1.124 0.949	26.836 15.822 36.459	0.000 ** 0.000 ** 0.000 **

Note. ** *p* < 0.01

**Table 5:** Means (centroids), standard deviations, and between-group comparisons for architectural elements (N = 231).

Architectural Variables	Assimilative N = 82 Value ^a^	Endopathic N = 90 Value	Additive N = 59 Value	*F*	*p*
S2. Complex geometric shapes	2	1	2	2.542	0.081 ^†^
P2. Proportion relative to the human scale	2	2	2	0.367	0.693
G1. Gravity of the space with respect to the ground	3	2	3	2.398	0.093 ^†^
G2. Weight of the supporting structure	2	3	2	2.957	0.054 ^†^
R2. Position of a set of elements in relation to each other	2	1	1	9.935	0.000 **
R3. Position of an object with respect to the whole space	7	2	2	497.568	0.000 **
L1. Natural daylight/openings	3	4	3	3.167	0.044 *
L2. Artificial light/temperature of light	1	2	2	4.735	0.010 *
C2. Color saturation/intensity	2	2	2	0.022	0.979
C3. Color range/temperature	3	3	3	2.912	0.056 ^†^

Note. ^a^ = Selected figure. ** *p* < 0.01, * *p* < 0.05, † *p* < 0.10.

## Data Availability

Data are available upon request.
